# The serine/threonine protein kinase MpSTE1 directly governs hyphal branching in *Monascus* spp.

**DOI:** 10.1007/s00253-024-13093-7

**Published:** 2024-03-06

**Authors:** Yali Duan, Xizhu Chen, Tingya Wang, Mu Li

**Affiliations:** 1 Key Laboratory of Environment Correlative Dietology, College of Food Science and Technology, Hubei International Scientific and Technological Cooperation Base of Traditional Fermented FoodsHuazhong Agricultural UniversityHubei Province, Wuhan, 430070 China; 2 College of Food Science and Technology, Huazhong Agricultural University, Hubei Province, Wuhan, 430070 China

**Keywords:** Filamentous fungi, Hyphal branching, Protein kinase, Signaling pathway, Secondary metabolites

## Abstract

**Abstract:**

*Monascus* spp. are commercially important fungi due to their ability to produce beneficial secondary metabolites such as the cholesterol-lowering agent lovastatin and natural food colorants azaphilone pigments. Although hyphal branching intensively influenced the production of these secondary metabolites, the pivotal regulators of hyphal development in *Monascus* spp. remain unclear. To identify these important regulators, we developed an artificial intelligence (AI)–assisted image analysis tool for quantification of hyphae-branching and constructed a random T-DNA insertion library. High-throughput screening revealed that a STE kinase, MpSTE1, was considered as a key regulator of hyphal branching based on the hyphal phenotype. To further validate the role of MpSTE1, we generated an *mpSTE1* gene knockout mutant, a complemented mutant, and an overexpression mutant (OE::*mpSTE1*). Microscopic observations revealed that overexpression of *mpSTE1* led to a 63% increase in branch number while deletion of *mpSTE1* reduced the hyphal branching by 68% compared to the wild-type strain. In flask cultures, the strain OE::*mpSTE1* showed accelerated growth and glucose consumption. More importantly, the strain OE::*mpSTE1* produced 9.2 mg/L lovastatin and 17.0 mg/L azaphilone pigments, respectively, 47.0% and 30.1% higher than those of the wild-type strain. Phosphoproteomic analysis revealed that MpSTE1 directly phosphorylated 7 downstream signal proteins involved in cell division, cytoskeletal organization, and signal transduction. To our best knowledge, MpSTE1 is reported as the first characterized regulator for tightly regulating the hyphal branching in *Monascus* spp. These findings significantly expanded current understanding of the signaling pathway governing the hyphal branching and development in *Monascus* spp. Furthermore, MpSTE1 and its analogs were demonstrated as promising targets for improving production of valuable secondary metabolites.

**Key points:**

• *MpSTE1 is the first characterized regulator for tightly regulating hyphal branching*

• *Overexpression of mpSTE1 significantly improves secondary metabolite production*

• *A high-throughput image analysis tool was developed for counting hyphal branching*

**Supplementary Information:**

The online version contains supplementary material available at 10.1007/s00253-024-13093-7.

## Introduction

Filamentous fungi are extensively utilized in industrial processes for the production of enzymes, organic acids, antibiotics, pigments, and medicines via fermentation (Liao et al. 2007). *Monascus* species are well-known producers of various polyketide secondary metabolites such as monacolins and azaphilone pigments (Chen et al. 2015). Monacolin K, originally isolated from *Monascus ruber* (Endo 1979), is identical to the drug lovastatin independently discovered in *Aspergillus terreus* (Alberts et al. 1980). As natural food colorants, *Monascus* azaphilone pigments have been widely used in Asia for centuries and possess a broad range of biological functions, such as anticancer, antimicrobial, and antiobesity bioactivities (Duan et al. 2022c; Huang et al. 2023). *Monascus*-fermented products are estimated to be consumed by over one billion people daily, especially across Southeast Asia (Liu et al. 2020).

In submerged culture, many filamentous fungi including *Monascus* spp. grow as multicellular hyphal aggregates called pellets (Zhang and Zhang 2016). Pellet formation and structure are influenced by the external environment as well as genes regulating fungal development (Dynesen and Nielsen 2003b). Furthermore, hyphal morphology strongly impacts metabolite biosynthesis (Harris 2019; Veiter et al. 2018). For instance, small pellets formed by *A. terreus* create favorable conditions for improved lovastatin production while dispersed filaments of *Aspergillus nidulans* enhance penicillin production (Moore and Bushell 1997; Saberi et al. 2020). Recently, it was reported that pH and inoculum size were critical factors governing pellet formation of* Monascus purpureus* (Zhang et al. 2023a, b). However, the regulatory mechanisms underlying pellet formation remain unclear (Posch et al. 2013), hindering efforts to improve fermentation performance in *Monascus* and other fungi.

A key determinant of fungal pellet density is hyphal branching (Steinberg et al. 2017). Individual spores germinate to produce multiple hyphae comprising a mycelial network. Because nutrients available at actively growing hyphal tips support the development and sporulation, maximizing the number of hyphal tips via branching provides growth/reproductive advantages (Harris 2008). Lateral hyphal branching is initiated through polarity establishment, allowing iterative production of hyphae (Dynesen and Nielsen 2003a).

While recent studies have uncovered several proteins regulating hyphal branching in fungi (Martin and Chang 2005; Park and Bi 2007), many key regulators and mechanisms remain to be elucidated. Potential pathways integrating hyphal branching with growth include the protein kinase A (PKA) and target-of-rapamycin (TOR) pathway. Both PKA and TOR coordinate budding and cell growth in yeast models (Broach 2012). Moreover, branching on hyphae of filamentous fungi is linked to nuclear division (Fiddy and Trinci 1976) and involves cell-cycle element like cyclin-dependent kinase (CDK) (Lin and Momany 2004). These findings revealed a genetic network regulating branch initiation, but there are substantial knowledge gaps regarding the precise functions of pathway in filamentous fungi (Harris 2019).

The objective of this study was to elucidate key proteins regulating hyphal branching in *Monascus* species. A random T-DNA insertion library was generated and subjected to high-throughput phenotypic screening which identified 18 proteins significantly altering the hyphal branching. A serine/threonine protein kinase (STE), MpSTE1, exhibited the most pronounced effect on branching. Through gene knockout and overexpression, MpSTE1 was found to positively regulate hyphal branching, fungal growth, and secondary metabolite production. Subsequent phosphoproteomic profiling revealed MpSTE1 phosphorylated 7 downstream proteins involved in MAPK signaling, cytoskeleton, and cell division. For the first time, we elucidated the STE-MAPK cascade regulated the hyphal branching of *M. purpureus* by protein phosphorylation.

## Materials and methods

### Strains and chemical regents

Wild-type (WT) *M. purpureus* strain HJ11 used in this study was obtained from the China General Microbiological Culture Collection Center (CGMCC), accession number 25474 (Duan et al. 2022c). The strains OE::*mpSTE1*, CΔ*mpSTE1*, and Δ*mpSTE1* were deposited in CGMCC with accession numbers 27584, 27,585, and 27,586, respectively. *Agrobacterium tumefaciens* LBA1100 (Biobw, Beijing, China) served as the plasmid DNA donor for genetic transformation of *M. purpureus*, and *Escherichia coli* TOP10 was utilized for recombinant plasmid construction. Hygromycin B used for selection of transformants was purchased from Sigma-Aldrich (St. Louis, MO, USA). The restriction enzymes and T4 DNA ligase were acquired from Thermo Fisher Scientific (Waltham, MA, USA). Unless indicated, all other chemicals and reagents were obtained from Sigma Co. Ltd (Shanghai, China).

### Cultivation and medium

*M. purpureus* strains were pre-cultivated on potato dextrose agar (PDA) medium for 8 days. Mature mycelia were then washed with sterile water to obtain a conidial suspension for inoculation. The conidial solution was standardized to 2 × 10^5^ conidia/mL prior to inoculation of the production medium. Lovastatin and azaphilone production was determined after cultivation in 250-mL flasks containing 50 mL modified Czapek-Dox (mCD) medium for 10 days at 30 °C with shaking at 150 rpm. For microscopic observation of hyphal branches, the nascent hypha was gently transferred onto microscope slides after 18-h cultivation of conidia in mCD medium. The number of hyphal branches was observed and recorded using a microscope. The mCD medium consisted of (g/L) glucose 30, NaNO_3_ 10, KH_2_PO_4_ 5, Na_2_HPO_4_ 3, MgSO_4_ 0.1, CaCl_2_ 0.1, ZnSO_4_·7H_2_O 0.1, FeSO_4_·7H_2_O 0.1, CoSO_4_·7H_2_O 0.05, CuSO_4_·5H_2_O 0.02, and MnSO_4_·H_2_O 0.01.

### Random mutagenesis library

The T-DNA plasmid was constructed by modifying the shuttle vector pCB301. The T-DNA insertion cassette consisted of the *tef1* promoter (P_*tef1*_) driving expression of a hygromycin B resistance gene (*hyg*) followed by the *tef1* terminator (T_*tef1*_). The physical map of T-DNA insertion cassette was the same as the plasmid pTFCM previously described (Lv et al. 2016). This recombinant plasmid was then transformed into *A. tumefaciens* LBA1100. Transformation of *M. purpureus* HJ11 was performed using established *A. tumefaciens-*mediated (AMT) method as we previously described (Duan et al. 2022a). Putative hygromycin-resistant transformants were subjected to three rounds of selection on PDA medium containing 50 μg/mL hygromycin B prior to further analysis.

For high-throughput screening of the generated mutants, strains were cultivated in 96-well microplates (1 mL round deepwell) containing 0.5 mL of mCD medium. Plates were incubated at 28 °C with shaking at 350 rpm (3-cm diameter) for 5 days to obtain conidia. The conidia were collected by centrifugation and used to inoculate fresh 96-well plates containing 0.5 mL of fresh mCD medium per well. Following further incubation for 18 h under identical conditions, newly formed hyphae were transferred to microscope slides, visualized under a light microscope, and photographed for quantification of hyphal branching phenotypes.

### High-throughput analysis of hyphal branching

To enable high-throughput image analysis, a deep learning model was developed for automated quantification of hyphal branching. The convolutional neural network consisted of an encoder-decoder architecture implemented in Keras 2.0 with a TensorFlow backend (Kim et al. 2021). The encoder that contained three convolutional layers was followed by max pooling layers and activated by a rectified linear unit function. The decoder was composed of corresponding convolution and upsampling layers. Feature maps from the final convolutional layer was passed to a softmax classifier to generate pixel-wise predictions of hyphal branch regions. To obtain enough training data, image scale was augmented from 3500 original microscopy images by random horizontal/vertical shifts, rotations, scaling, and shear. This data augmentation procedure yielded an expanded dataset of ∼35,000 images which were further corrupted with random noise and blurring. The generated model was utilized for automated high-throughput quantification of hyphal branching morphology from the microscopic images of the mutant strains.

### Phosphoproteome analysis

To characterize the phosphoproteome, total protein was extracted from mycelia and subjected to enzymatic digestion coupled with phosphopeptide enrichment prior to tandem mass spectrometry. In brief, 10-day-old vegetative mycelia were washed three times with ice-cold PBS, mechanically lysed by high-pressure homogenization, and centrifuged to remove cellular debris. The resulting supernatant was reduced with 10 mM tris (2-carboxy(ethyl)phosphine) (TCEP) and alkylated using 40 mM 2-chloroacetamide (CAA). Protein digestion was performed by addition of trypsin and lysC for 12 h at 37 °C. Phosphopeptide enrichment was carried out as previously described (Zimman et al. 2010). Phosphopeptide enrichment was subsequently performed using established methods. Peptide fragments were fractionated by reverse-phase nanoLC (EASY-Spray column, 25 cm × 75 μm, PepMap C18) (Thermo Fisher Scientific, MA, USA) coupled to a Thermo Scientific EASY-nLC 1000 system (Thermo Fisher Scientific, MA, USA). Tandem mass spectrometry was carried out on a Thermo Q-Exactive instrument with technical duplicates for each sample. The resulting mass spectrum (MS) data were analyzed in MaxQuant 1.6.0.1 using the search engine Andromeda (Pinto et al. 2020) and UniProt reference proteome of *M. purpureus* strain NRRL 1596. Phosphorylation of serine, threonine, and tyrosine residues was defined as a variable modification. Comparative phosphoproteomic analysis of mutant strain between wild-type strain revealed differentially phosphorylated proteins for further investigation. Peptide fragmentation was carried out via higher-energy collisional dissociation (HCD). Raw mass spectrometric data were processed in MaxQuant (version 1.6.0.1) using the Andromeda search engine. Data were searched against a UniProt *M. purpureus* reference proteome (Zhang et al. 2023a, b), with phosphorylation of serine, threonine, and tyrosine residues defined as variable modifications. Label-free quantification (LFQ) was enabled with default parameters. Downstream statistical analyses were performed in Perseus to identify differentially phosphorylated proteins between wild-type and mutant strains. The mass spectrometry proteomics data have been deposited to the ProteomeXchange via the iProX database partner repository (accession number: PXD048208).

### Quantitation of lovastatin and azaphilone

Extraction and quantification of lovastatin and azaphilone was performed as we previously described (Duan et al. 2022b, 2023). After 10 days of cultivation, *M. purpureus* mycelia were disrupted by high-pressure homogenization using a French press. The resultant paste was then acidified to pH < 4.0 and extracted three times with 80% (v/v) ethanol. The pooled extracts were clarified by centrifugation, evaporated to dryness by nitrogen gas, and resuspended in methanol for high-performance liquid chromatography analysis. Metabolite quantification was conducted on a Shimadzu Prominence system comprised of an autosampler (SIL-20A) (Shimadzu, Shanghai, China) and a photodiode array detector (SPD-M20A) (Shimadzu, Shanghai, China). Commercial standards were used to generate calibration curves for lovastatin and azaphilone based on integrated peak areas from three independent experiments.

### Quantitative real-time PCR

Total RNA extraction was performed using TRIzol reagent after mycelia were frozen and ground to a fine powder. cDNA was synthesized from the isolated RNA by PrimeScript reverse transcriptase (TaKaRa, Dalian, China). qRT-PCR was conducted in triplicate reactions using SYBR Premix Ex Taq (TaKaRa, Dalian, China) on an Applied Biosystems 7500 Fast instrument (Thermo Fisher Scientific, MA, USA) with the following cycling conditions: initial denaturation at 95 °C for 10 s; 40 cycles of 95 °C for 3 s; and 60 °C for 25 s. Target mRNA abundance was quantified by the cycle threshold (CT) method (2^−ΔΔCT^) using β-actin for normalization.

## Results

### Constructing a high-throughput hyphae-branching mutant screening strategy

**Fig. 1:**
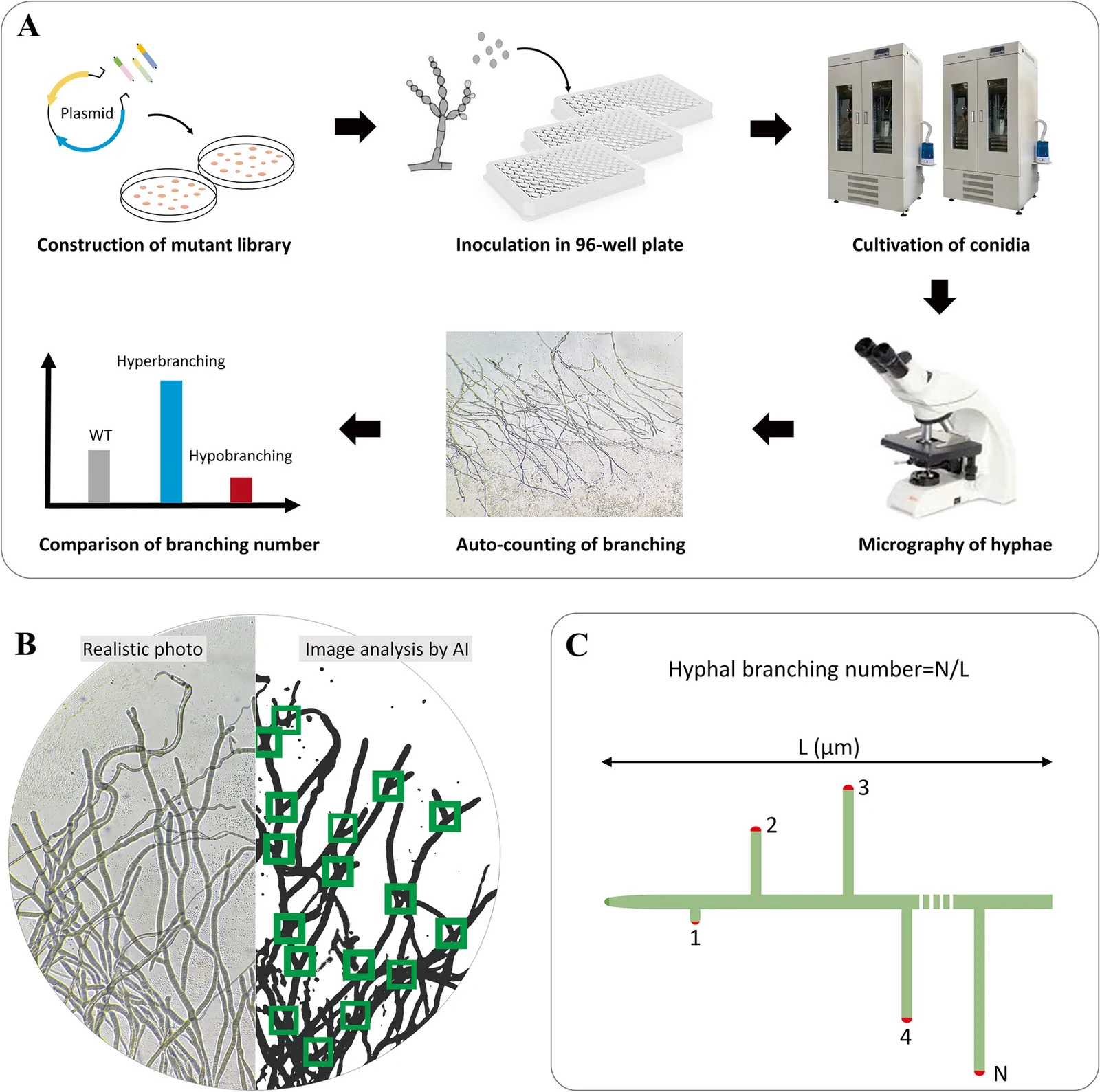
Schematic overview of a high-throughput hyphae-branching mutant screening strategy based on a deep learning model for micrography analysis. **A** After the cultivation of conidia obtained from *M. purpureus* mutant strains was performed in 96-well plates, the nascent hyphae were transferred to microscope slides. The micrographs of hyphal branching were captured and stored. The hyphal branching levels of various mutants were automatically evaluated by the deep learning model HBMD. **B** Visual recognition of hyphal branching points in micrographs by HBMD. **C** Schematic of determining the level of hyphal branching

There are no studies on genes directly related to hyphal branching in *Monascus* spp., so we decided to construct a random T-DNA insertion library to screen for genes affecting the hyphal branching phenotype (Fig. 1). However, no high-throughput screening approach for this phenotype was previously available. We therefore established a strategy to rapidly quantify the hyphal branching of numerous mutants (Fig. 1A). The workflow entailed cultivating conidia in 96-well plates, transferring hyphae to microscope slides, and micrography of hyphal branching. To rapidly analyze the images, a deep learning model (hyphal branching model (HBMD)) was developed to automatically evaluate the branching number (Fig. 1B). The accuracy of HBMD on the test images was close to 97%, indicating that our model achieved high recognition accuracy and confidence (Supplemental Fig. S1). The level of hyphal branching was defined as the lateral branch number per 100-μm hyphal length (Fig. 1C). On average, the deep learning model took only 2.5 min in processing 10 micrographs which were obtained for each mutant strain. Consequently, the entire process from conidia inoculation to branching quantification required only 24 h, therefore greatly improving screening efficiency.

### Screening a random T-DNA insertion library for genes involved in hyphal branching

To identify genes regulating hyphal branching in *M. purpureus*, we constructed a T-DNA insertion library to generate random mutagenesis. The principle of our T-DNA insertion library was that T-DNA fragments were randomly inserted into any site (Kemppainen et al. 2008) in the *M. purpureus* genome to randomly inactivate gene functions. We hypothesized that mutants with defects in hyphal branching formation would display a large deviation in hyphal morphology compared to the wild-type strain.

**Fig. 2:**
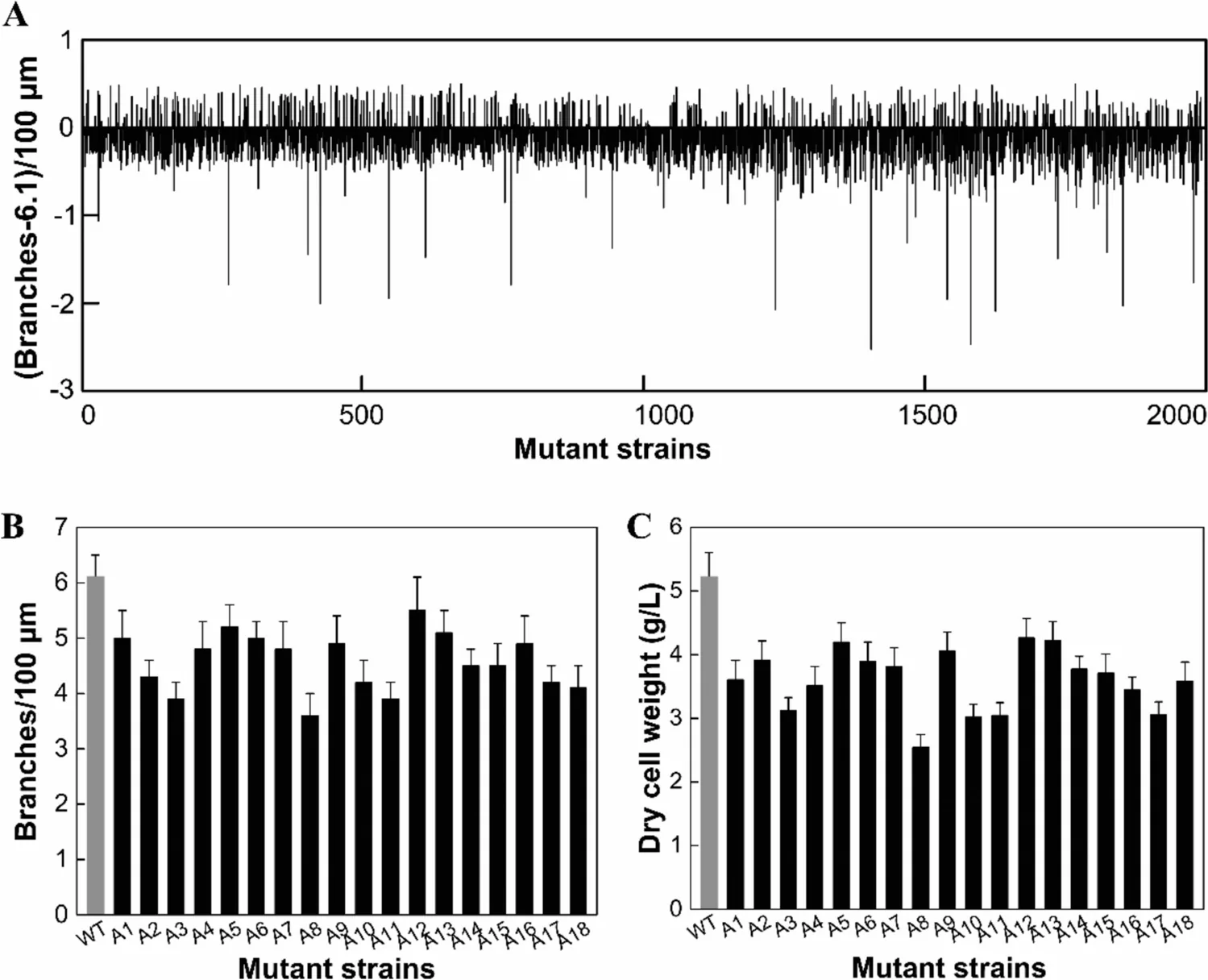
Screening and characterization of hyphae-branching mutant strains. **A** High-throughput screening of branching numbers of mutants in the T-DNA insertion library. The branching numbers of 1992 mutant strains were determined by the HBLM model after cultivation in 96-well microplates. The branching number was calculated by mutant strain plus wild-type strain. **B** Hyphae-branching number of 18 mutant strains. Eighteen strains were selected from the high-throughput screening result due to their reducing branching numbers. *M. purpureus* strains were cultivated in a flask for precise determination of branching number. **C** Dry cell weight of 18 mutant strains. Error bars represent the SD of three experiments

Approximately 2000 transformants were generated in the random T-DNA insertion library and then cultivated in batches in 96-well plates for 18 h. Microscopic images were captured and analyzed by our automated model HBMD. The number of hyphal branching for each transformant was evaluated and normalized to the wild-type control. The screening result uncovered 18 mutants with significantly reduced branching numbers (Fig. 2A). In Fig. 2A, we subtracted the number of branches of each transformant from 6.1, where 6.1 is the average number of branches per 100-μm distance. To validate the screening result, conidia of the 18 strains were inoculated into 100-mL shake flask cultures for 18 h before manual quantification of branching. Three transformants, A3, A8, and A11, produced the lowest branching numbers (Fig. 2B). We further cultivated these strains in shake flasks for 10 days, and the biomass of these three strains also remained the lowest among all 18 strains (Fig. 2C). Strain A8, which displayed the lowest branching, was selected for further characterization.

Combination of whole-genome sequencing of strain A8 and whole-genome alignment for the wild-type and A8 strains revealed that *M. purpureus* strain A8 contained the sole disruption of the *mpSTE1* gene. Bioinformatic analysis predicted that MpSTE1 (protein ID e_gw1.36.93.1) harbored a conserved STKc catalytic domain (Supplemental Fig. S2), mediating the transfer of the γ-phosphoryl group from ATP to serine/threonine residues on protein substrates (Ma et al. 2021). We therefore proposed that MpSTE1 is a serine/threonine protein kinase (STE) and regulates the hyphal branching in *M. purpureus* through signal transduction.

### Gene knockout and overexpression of the gene mpSTE1

**Fig. 3:**
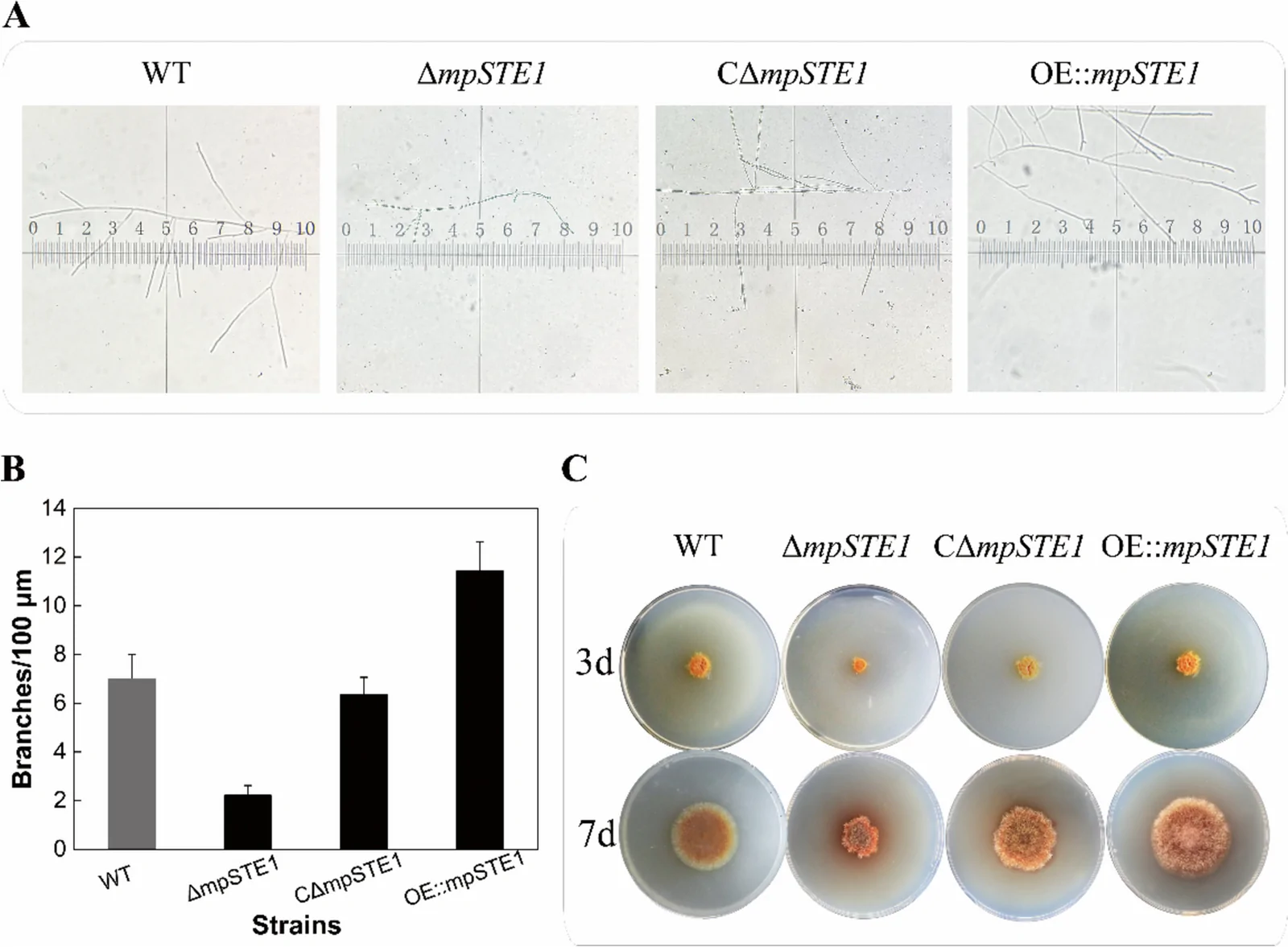
*M. purpureus mpSTE1* gene mutant strains displayed altered hyphal branching phenotype. **A** Representative micrographs of WT, Δ*mpSTE1*, CΔ*mpSTE1*, and OE::*mpSTE1* strains grown in mCD medium. Eighteen-hour-old hyphae were transferred to slides for micrography. Scale bars represent 20 µm. **B** Branching quantification ofΔ*mpSTE1*, CΔ*mpSTE1*, and OE::*mpSTE1* strains and WT control in the experiment conducted in the same as (**A**). Three micrographs were acquired from three flasks for each strain (*n* = 3). **C** Colony morphology of WT and Δ*mpSTE1*, CΔ*mpSTE1*, and OE::*mpSTE1* strains. The strains were cultivated on PDA plates at 28 °C for 7 days

To further validate the role of MpSTE1 in hyphal branching of *M. purpureus*, we employed the *Agrobacterium*-mediated transformation method to generate *mpSTE1* gene knockout (Δ*mpSTE1*), complemented (CΔ*mpSTE1*), and overexpression (OE::*mpSTE1*) mutants. The result of relative expression of the *mpSTE1* gene in these strains indicated that several strains were successfully constructed (Supplemental Fig. S3). Significant differences in the hyphal branching numbers were observed among the mutants. Compared to the WT strain, the strain Δ*mpSTE1* displayed less number of hyphal branching while the strain OE::*mpSTE1* showed increased branching number (Fig. 3A). The mutant Δ*mpSTE1* displayed a 40% reduction in lateral branching number relative to the WT strain. Conversely, the strain OE::*mpSTE1* exhibited a 120% increase in branching number (Fig. 3B). After cultivation on PDA medium, the diameter of OE::*mpSTE1* colony was markedly larger than those of WT, Δ*mpSTE1* and CΔ*mpSTE1* strains, whereas the radial growth of strain Δ*mpSTE1* was diminished. The strain CΔ*mpSTE1* showed a colony size similar to that of the WT strain. Taken together, these phenotypic impacts demonstrated MpSTE1 positively regulated hyphal branching and development in *M. purpureus*.

### Overexpression of mpSTE1 exhibited rapid growth and high production of lovastatin and azaphilone pigments

**Fig. 4:**
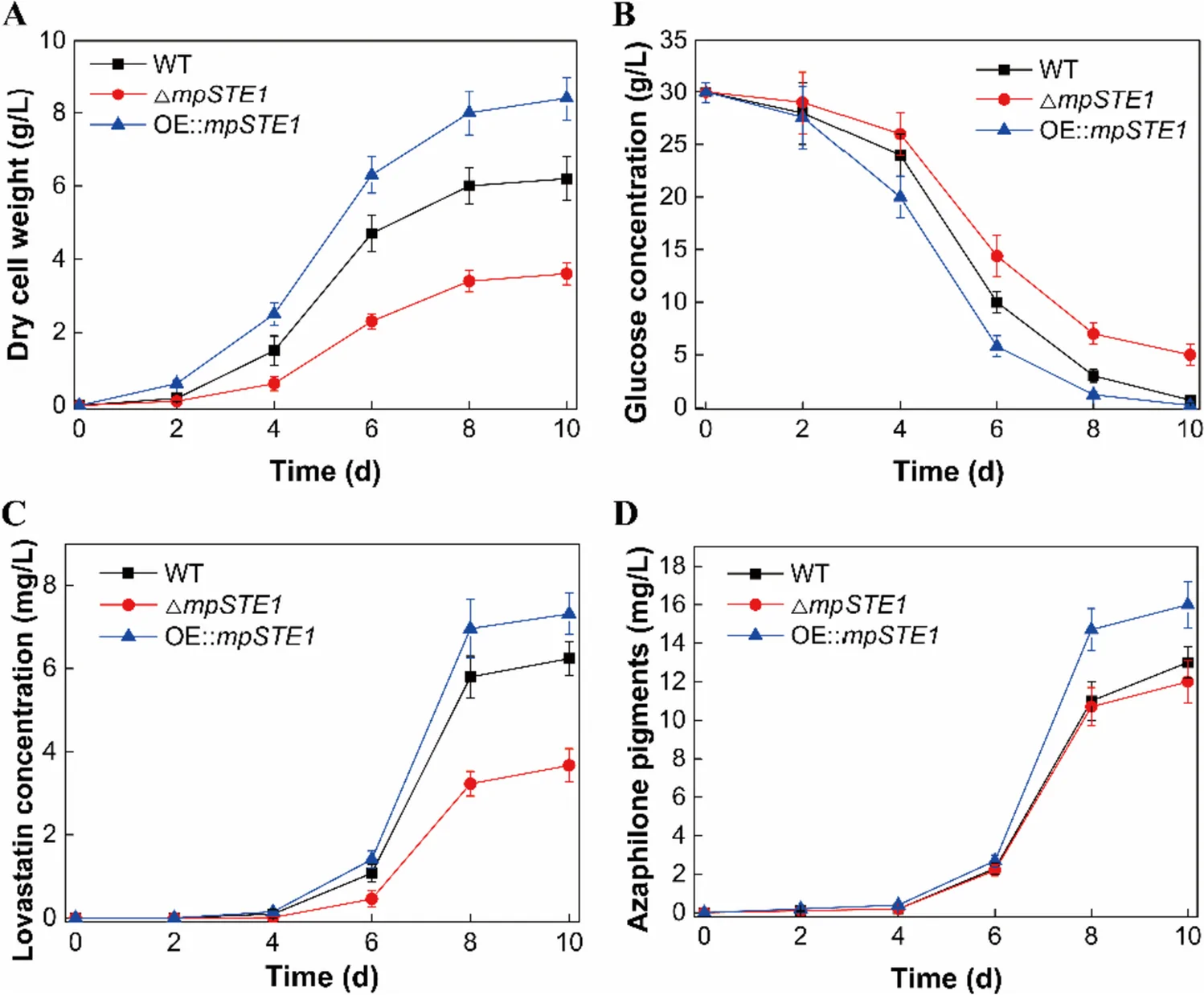
Comparison of growth and secondary metabolite production between WT and *mpSTE1* mutant strains. Growth (**A**), glucose consumption (**B**), lovastatin (**C**), and azaphilone pigment (**D**) curves of the WT, Δ*mpSTE1*, and OE::*mpSTE1* strains. The value was evaluated in triplicate for each strain, and the average values and standard deviations were calculated based on the results

As OE::*mpSTE1* colonies grew faster than WT or ΔmpSTE1 strains (Fig. 3C), we proposed that overexpression of *mpSTE1* might also promote the production of secondary metabolites lovastatin and azaphilone pigments. To verify this hypothesis, WT, Δ*mpSTE1*, and OE::*mpSTE1* strains were cultivated in 250-mL flasks containing 50 mL mCD medium for 10 days. Fermentation kinetics and metabolite levels were assessed over time. The OE::*mpSTE1* strain displayed a high growth, resulting in a final dry cell weight (DCW) of 8.4 g/L, markedly higher than those of the WT and Δ*mpSTE1* strains (Fig. 4A). The strain OE::*mpSTE1* also consumed glucose faster than those of the WT and Δ*mpSTE1* strains, which was consistent with the growth curves (Fig. 4B). More importantly, the strain OE:*mpSTE1* yielded higher lovastatin (7.3 mg/L) and azaphilone pigments (16.0 mg/L) than those of the WT and Δ*mpSTE1* strains (Fig. 4C and D). In addition, the Δ*mpSTE1* strain exhibited lower biomass and secondary metabolite production than those of the WT strain. Together, these results demonstrated that MpSTE1-mediated hyphae-branching enhanced both fungal growth and secondary metabolite production.

### Identification of MpSTE1 targets by phosphoproteomic analysis

Considering that MpSTE1 is a protein kinase, MpSTE1 mediates the phosphorylation of downstream proteins which control hyphal branching in *M. purpureus*. The phosphoproteomes of the wild-type (WT) and Δ*mpSTE1* strains were compared to pinpoint proteins specifically phosphorylated in an MpSTE1-dependent manner. Since the gene *mpSTE1* was disrupted in the mutant, its targets should not undergo phosphorylation compared to the WT strain. The data were filtered for phosphorylated peptides present in all WT replicates but absent in all Δ*mpSTE1* replicates. We identified 7 proteins, with distinct phosphorylation sites, as putative MpSTE1 targets (Table 1). These proteins are uncharacterized in *M. purpureus* yet homologous to proteins with established functions in other fungi.

**Table 1:** Putative target proteins and target sites of MpSTE1 identified in this study

No	Name	Protein ID	Phosphorylated peptide	Protein description	Biological process
1	MpAPC	CE23238_2004	WPDHDS**T**^**246**^WG	Anaphase promoting complex	Cell division
2	MpCDK	CE387899_9144	NAVNLPATP**T**^**660**^GPRD	Cyclin-dependent kinase	Cell division
3	MpCSN	gw1.70.23.1.83	PATNPP**T**^**159**^INV	COP9 signalosome	Signal transduction
4	MpCKI	1.C_210035	QKKP**T**^**430**^SDPKGR	Cyclin-dependent kinase inhibitor	Cell division
5	MpSPT	348v1rpkm516.51	GSEKDVK**T**^**238**^ADS	Septin	Cytoskeleton
6	MpTPR	pg.37_#_2	ANNVG**T**^**60**^PDQDS	Transcriptional repressor	Translation
7	MpMGA1	gw1.20.35.1	LRSRVK**T**^**181**^TGIT	GPCR α subunit	Signal transduction

Our mass spectrometry–based phosphoproteome result supported that several proteins involved in cell division, signal transduction, and cytoskeletal were phosphorylation targets of MpSTE1. Many studies have demonstrated that STEs regulated downstream effectors by phosphorylation of threonine residue (Ghorai et al. 2018). Notably, both a cyclin-dependent kinase (MpCDK) and its corresponding inhibitor (MpCKI) were phosphorylated by MpSTE1, implying concurrent modulation of MpCDK and MpCKI to regulate cell cycling and hyphal development in *M. purpureus*. The remaining 5 proteins, including an anaphase promoting complex (MpAPC), a COP9 signalosome (MpCSN), a septin (MpSPT), a transcriptional repressor (MpTPR), and a G protein–coupled receptor α subunit (MpMGA1), were also subjected to MpSTE1 phosphorylation.

Phosphorylated peptides that were identified in the WT in every replicate but in none of the replicates of the Δ*mpSTE1* mutant were considered as the putative target substrates of MpSTE1. The phosphorylated amino acid is indicated as bold letter. *GPCR*, G protein–coupled receptor.

## Discussion

Hyphal branching is an essential feature underlying the development of fungi (Harris 2008). The process of forming a hyphal branch can be generally divided into four steps. The first step corresponds to the period during which the morphogenetic machinery (i.e., cytoskeleton and vesicle) is recruited to the incipient branch site. The second step refers to the period during which the morphogenetic machinery generates a stable polarity axis directing the emergence of the new branch. The third and final steps represent the period during which the new hyphal tip matures and attains its maximal extension rate (Seiler and Plamann 2003). A study of the mechanism of hyphal branching provides not only a better understanding of morphological development of filamentous fungi, but also a significant application in the fermentation industry (Ziv et al. 2009).

**Fig. 5:**
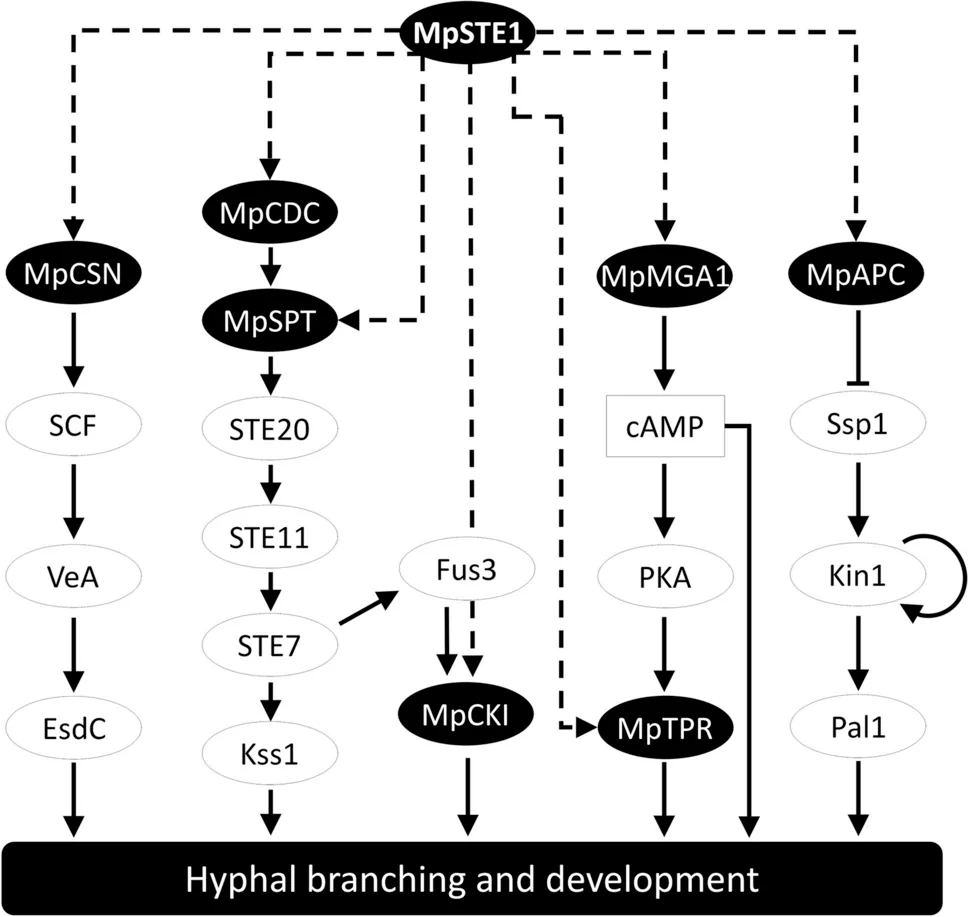
Proposed MpSTE1-mediated signaling pathway during response to hyphal branching in *M. purpureus*. The identified targets of MpSTE1 in *M. purpureus* are in black circles. Components that have been functionally characterized in other fungi are in hollow circles (Gil-Sánchez et al. 2022; Han et al. 2008; Zhao et al. 2007; Lee et al. 2018). Dashed lines represent direct phosphorylation regulation by MpSTE1

In this study, we identified MpSTE1 as a serine/threonine protein kinase in *M. purpureus* HJ11 from a random mutagenesis library. Both knockout and overexpression of *mpSTE1* markedly altered hyphae-branching pattern (Fig. 3), growth kinetics, and secondary metabolite production (Fig. 4). Phosphoproteomic analysis revealed MpSTE1-dependent phosphorylation of 7 distinct proteins involved in cell division, cytoskeleton, and signal transduction (Table 1). Based on published literature, we proposed a signaling cascade, including 8 proteins, in regulating hyphal branching in *M. purpureus* (Fig. 5).

A proposed MpSCN-SCF-VeA-EsdC sequential signaling may alter hyphae-branching development in *M. purpureus* under the regulation of MpSTE1 (Fig. 5). Recent studies highlighted the role of the COP9 signalosome (CSN) during fruiting body formation in the sexual reproduction of *A. nidulans* (Busch et al. 2007). Subsequent findings revealed that SCF E3 ubiquitin ligase- and CSN-mediated proteasome degraded the regulator VeA in aerial hyphae, which caused sexual differentiation (Gil-Sánchez et al. 2022; Jonkers and Rep 2009). Additionally, a glycogen-binding protein EsdC, which was identified downstream of VeA, modulated early hyphal development process (Han et al. 2008).

A sequential phosphorylation process (MpCDK-MpSPR-STE20-STE11-STE7-Kss1 and -STE7-Fus3-MpCKI) was proposed to form a regulatory pathway that regulated hyphae-branching development in *M. purpureus*. Canonical cyclin-dependent kinases (CDKs) and their interacting partners (CKIs) formed a protein machine mediating cell cycle progression in eukaryotes (Yue et al. 2017). In our study, the CDK MpCDK might mediated the phosphorylation of a septin, MpSPT, which likely modified cyclin binding to impact cell division (Campanella et al. 2022; Douglas et al. 2005; Yue et al. 2017). Subsequently, activation of the STE20-STE11-STE7-Kss1 module transmits biological signals to mediate fungal hyphae development (Chou et al. 2006; Zhao et al. 2007) (Fig. 5). In a branch of this pathway, STE7 inhibited the CKI protein MpCKI by phosphorylation through the Fus3 MAPK pathway (Bhattacharyya et al. 2006). These results further highlighted these cell-cycle elements for signaling networks involved in fungal morphogenesis (Halder et al. 2021).

G protein–coupled receptor (GPCR) pathways enable fungi to sense environmental signals to coordinate cellular processes, metabolism, and morphogenesis (Brown et al. 2018). Herein, a GPCR α subunit, MpMGA1, might be phosphorylated by MpSTE1 in *M. purpureus*. MpMGA1 likely controlled the cyclic AMP (cAMP) signaling, which played established roles in fungal hyphae development (Harashima and Heitman 2002). The adenylate cyclase, activated by G protein, triggered the activation of protein kinase A (PKA) in a cAMP-dependent manner. Then PKA phosphorylated the downstream factors regulating growth and morphological transitions (Cullen and Sprague 2012). For instance, PKA might inhibit the transcriptional repressor MpTPR to alter gene expression programs underlying hyphal branching (Adnan et al. 2017) (Fig. 5).

MpAPC was a subunit of the anaphase promoting complex/cyclosome (APC/C), which is a conserved multimeric E3 ubiquitin ligase, regulating cell cycle and mitotic/meiotic progression (Cooper et al. 2000). The APC/C inhibited the function of Ssp1, resulting in the coordination of cytokinesis (Diamond et al. 2009). Ssp1 subsequently phosphorylated a cell polarity protein Kin1 activation loop to promote growth at cell tips. A MARK/PAR-1-related kinase Kin1 and a cell polarity protein Pal1 localized to actively growing hyphal tips and Kin1 autophosphorylated in addition to phosphorylating the protein Pal1 to promote growth at cell tips (Lee et al. 2018). Therefore, we deduced a multi-step signaling pathway (MpAPC/Ssp1/Kin1/Pal1) as part of a signaling network involved in cell polarity in *M. purpureus*.

## Supplementary Information

Below is the link to the electronic supplementary material.

Supplementary file1 (PDF 329 KB)

## Data Availability

All data generated or analyzed during this study are included in this published article or are available from the corresponding author on reasonable request.
