# Supplementary material for: The serine/threonine protein kinase MpSTE1 directly governs hyphal branching in Monascus spp
Source: Appl Microbiol Biotechnol. 2024 Mar 6;108:255. doi: 10.1007/s00253-024-13093-7 (PMC10917826; doi:10.1007/s00253-024-13093-7)
Supplement: Supplementary file 1 [file 253_2024_13093_MOESM1_ESM.pdf]

Title page

Applied Microbiology and Biotechnology

Supplementary Information

The serine/threonine protein kinase MpSTE1 directly governs hyphal branching in *Monascus* spp.

Yali Duan[1,2#], Xizhu Chen[1,2#], Tingya Wang[1,2#], Mu Li[1,2*]

[1] Hubei International Scientific and Technological Cooperation Base of Traditional Fermented Foods, Key Laboratory of Environment Correlative Dietology, College of Food Science and Technology, Huazhong Agricultural University, Wuhan, Hubei Province, 430070, China

[2] College of Food Science and Technology, Huazhong Agricultural University, Wuhan, Hubei Province, 430070, China

* Corresponding authors: Mu Li, limu@mail.hzau.edu.cn; Fax: +86-27-87282111

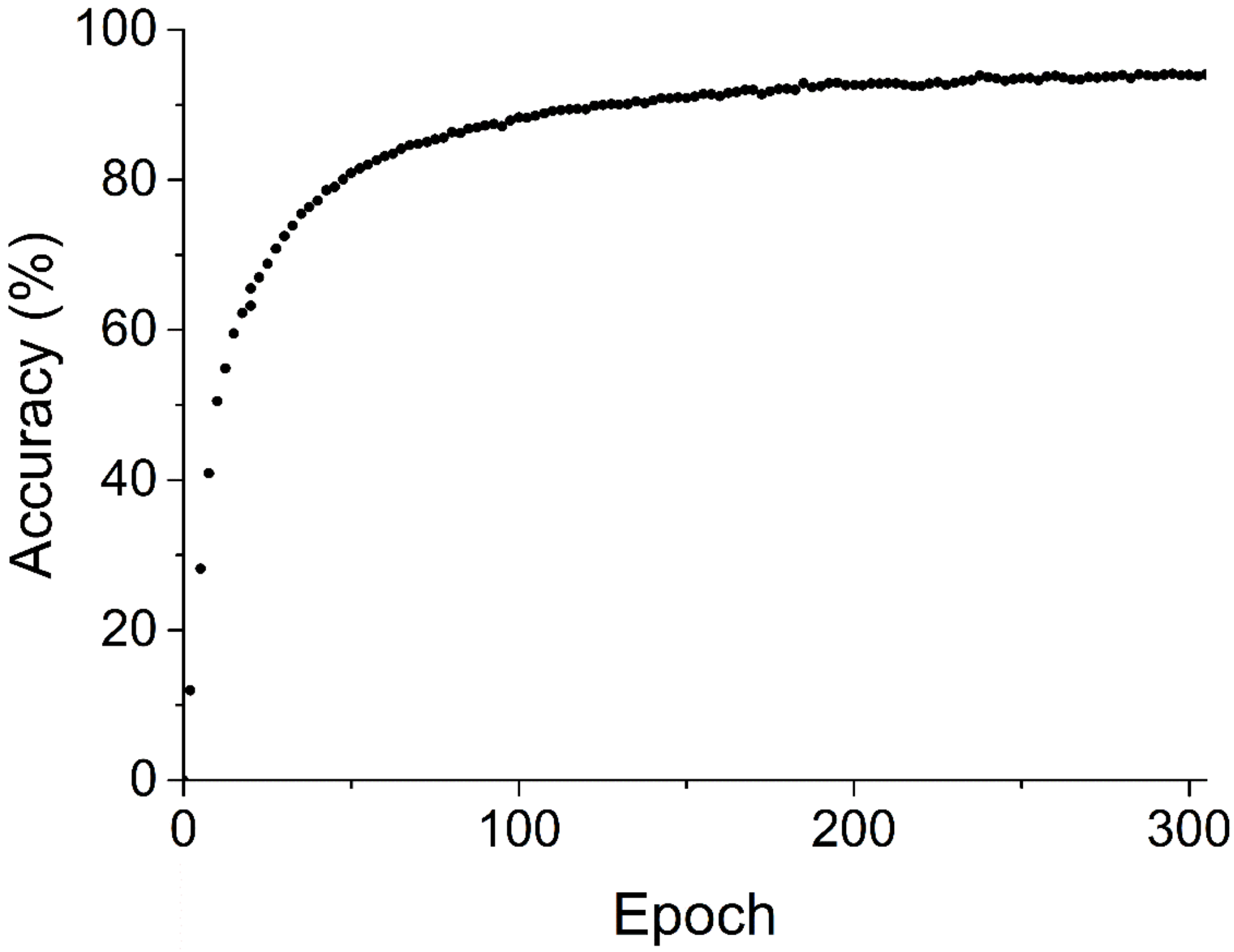

Fig. S1 Plot of accuracy on the training datasets over training epochs for quantification of hyphal branching in the micrograph by a deep learning model HBDL. The accuracy on the test set is close to 97%.

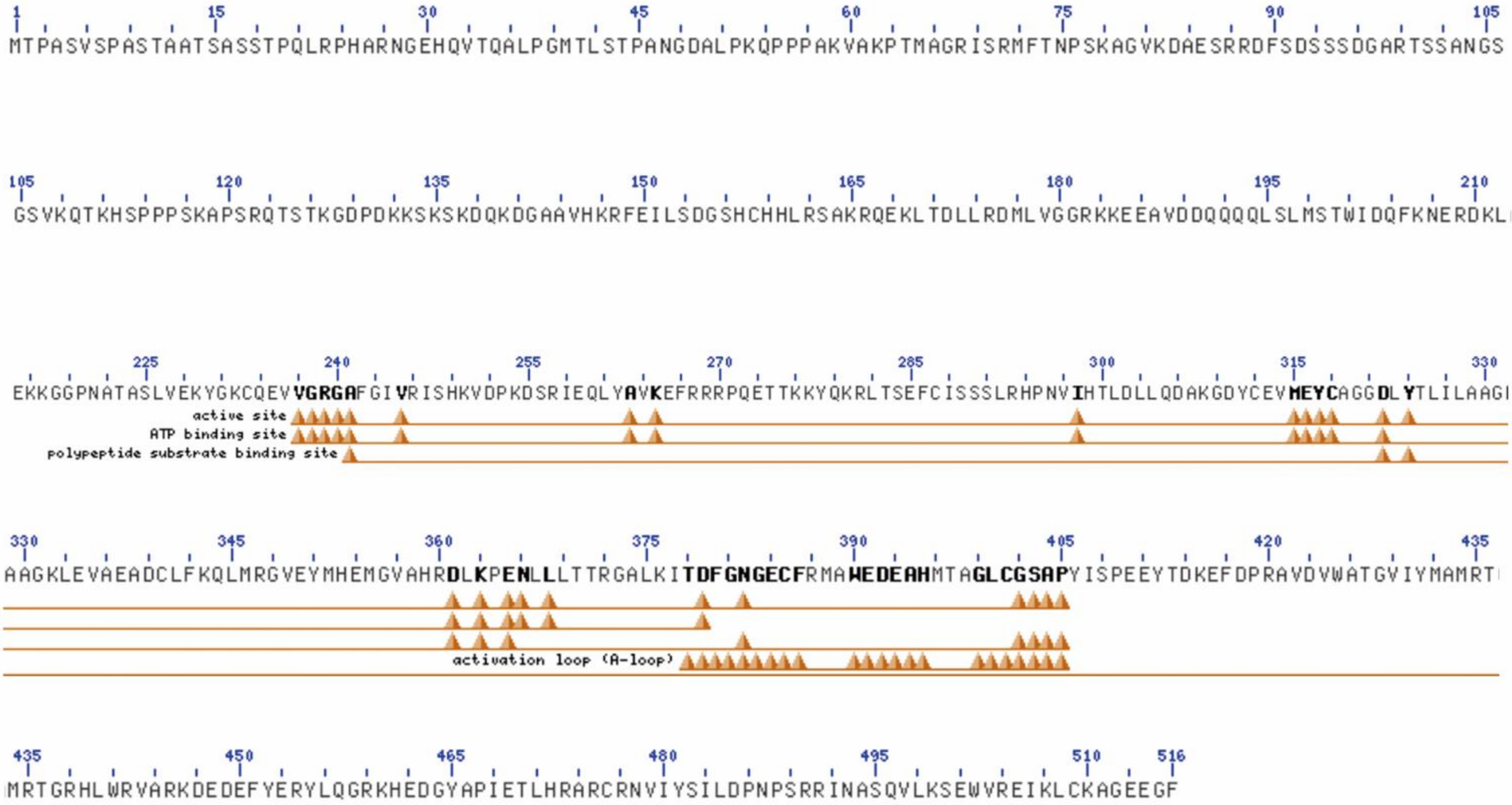

Fig. S2 Sequence and proposed active residues of MpSTE1. The conserved catalytic domain contained catalytic site, ATP binding site and polypeptide substrate binding site. The proposed site is indicated as bold letter.

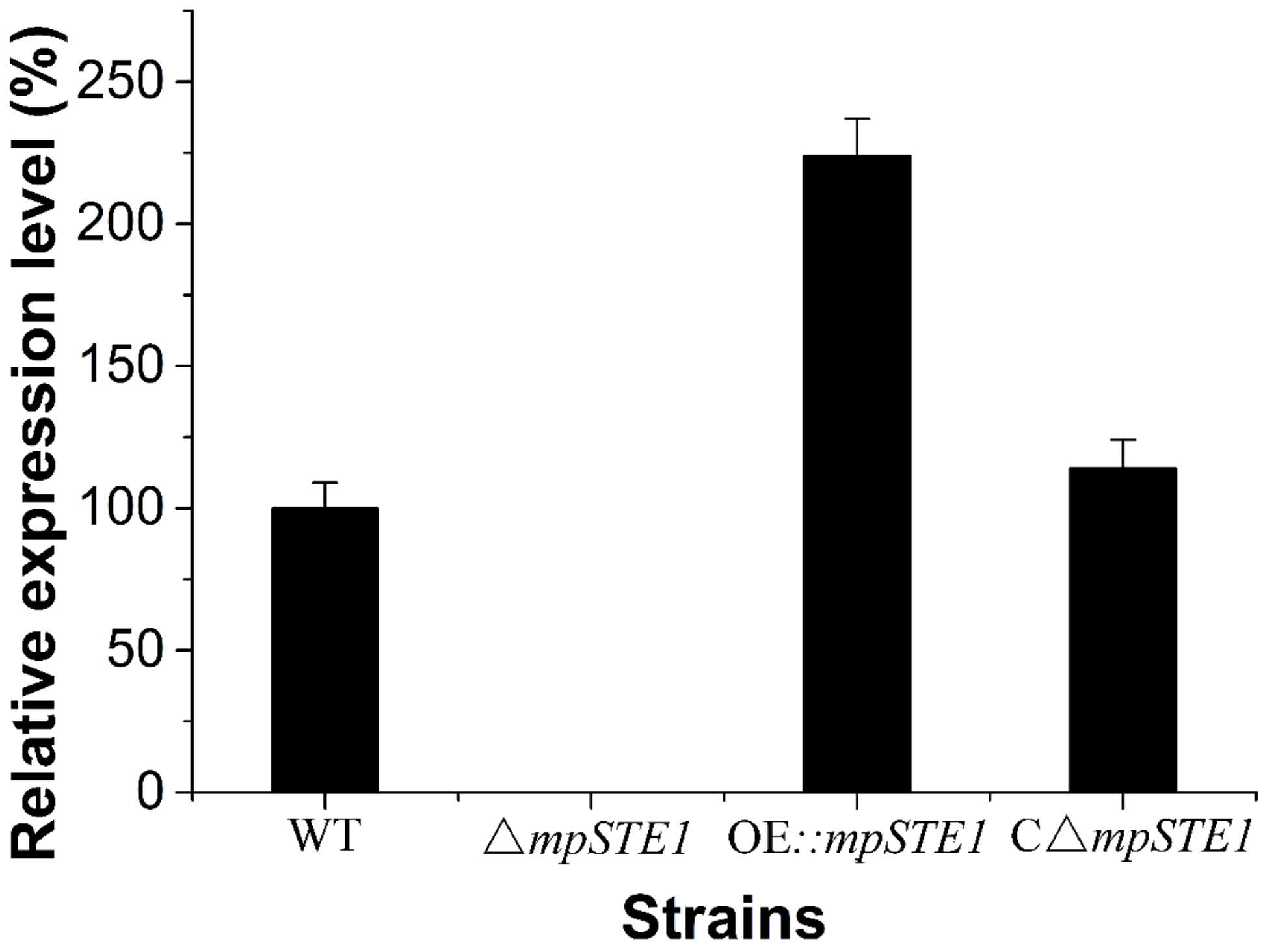

Fig. S3 The relative expression level of the wild-type strain, *mpSTE1* gene knockout (Δ*mpSTE1*), complemented (CΔ*mpSTE1*) and overexpression (OE::*mpSTE1*) mutants. The β-actin gene was used as an internal standard to normalize expression levels.
